# Characterization of Buriti (*Mauritia flexuosa*) Pulp Oil and the Effect of Its Supplementation in an In Vivo Experimental Model

**DOI:** 10.3390/nu14122547

**Published:** 2022-06-19

**Authors:** Gabriela Marcelino, Priscila Aiko Hiane, Arnildo Pott, Wander Fernando de Oliveira Filiú, Anderson R. L. Caires, Flavio S. Michels, Mário R. Maróstica Júnior, Nathalia M. S. Santos, Ângela A. Nunes, Lincoln C. S. Oliveira, Mário R. Cortes, Iriani R. Maldonade, Leandro F. Cavalheiro, Carlos Eduardo Domingues Nazário, Lidiani Figueiredo Santana, Carolina Di Pietro Fernandes, Fábio Juliano Negrão, Mariana Bento Tatara, Bernardo Bacelar de Faria, Marcel Arakaki Asato, Karine de Cássia Freitas, Danielle Bogo, Valter Aragão do Nascimento, Rita de Cássia Avellaneda Guimarães

**Affiliations:** 1 Graduate Program in Health and Development in the Central-West Region of Brazil, Federal University of Mato Grosso do Sul, Campo Grande 79070-900, Brazil; gabi19ac@gmail.com (G.M.); priscila.hiane@ufms.br (P.A.H.); lidi_lfs@hotmail.com (L.F.S.); nutricaroldipietro@gmail.com (C.D.P.F.); kcfreitas@gmail.com (K.d.C.F.); daniellebogo@hotmail.com (D.B.); aragao60@hotmail.com (V.A.d.N.); 2 Laboratory of Botany, Institute of Biosciences, Federal University of Mato Grosso do Sul, Campo Grande 79070-900, Brazil; arnildo.pott@gmail.com; 3 Pharmaceutical Science, Food and Nutrition Faculty, Federal University of Mato Grosso do Sul, Campo Grande 79070-900, Brazil; wander.filiu@gmail.com; 4 Optics and Photonics Group, Institute of Physics, Federal University of Mato Grosso do Sul, Campo Grande 79070-900, Brazil; andercaires@gmail.com (A.R.L.C.); santanamichels@gmail.com (F.S.M.); 5 Faculty of Food Engineering, University of Campinas, Campinas 13083-862, Brazil; mmarosti@unicamp.br (M.R.M.J.); nathmedina1@gmail.com (N.M.S.S.); 6 Program in Biotechnology, Dom Bosco Catholic University, Campo Grande 79117-900, Brazil; nunysnutri@yahoo.com.br; 7 Chemistry Institute, Federal University of Mato Grosso do Sul, Campo Grande 79070-900, Brazil; lincoln.oliveira@ufms.br (L.C.S.O.); mariocortess40@gmail.com (M.R.C.); lernfc@gmail.com (L.F.C.); cenazario@gmail.com (C.E.D.N.); 8 Laboratory of Food Sciences and Technology, Brazilian Agricultural Research Corporation (EMBRAPA Vegetables), Brasília 70770-901, Brazil; iriani.maldonade@embrapa.br; 9 Health Science Research Laboratory, Federal University of Grande Dourados, Dourados 79804-970, Brazil; fjnegrao@gmail.com (F.J.N.); marianabtatara@gmail.com (M.B.T.); 10 Diagnostic Medicine Laboratory—Scapulatempo, Campo Grande 79002-170, Brazil; bacelarfaria@gmail.com; 11 Medical School, Federal University of Mato Grosso do Sul, Campo Grande 79070-900, Brazil; marcel_arakakiasato@hotmail.com

**Keywords:** vegetable oils, fatty acids, animal models, antioxidant activity, interleukin-6

## Abstract

*Mauritia flexuosa* (Buriti) pulp oil contains bioactive substances and lipids that are protective against cardiovascular and inflammatory diseases. We performed physical and chemical analyses to verify its quality and stability. Buriti oil was stable according to the Rancimat test, presenting an induction period of 6.6 h. We evaluated the effect of supplementation with crude buriti oil and olive oil on metabolic parameters in 108 Swiss mice for 90 days. We investigated six groups: extra virgin olive oil (EVOO) 1 and 2 (1000 and 2000 mg/kg), buriti oil (BO) 1 and 2 (1000 and 2000 mg/kg), synergic (S) (BO1 + EVOO1), and control (water dose 1000 mg/kg). The animals were euthanized to examine their blood, livers, and fats. The supplementation did not interfere with food consumption, weight gain, and histological alterations in the liver. Group S showed the strongest relationship with the fractions HDL-c and non-HDL-c, indicating a possible cardioprotective effect. Moreover, we observed significantly higher IL-6 levels in the control, EVOO2, and BO1 groups than in the EVOO1 group. Resistin was also significantly higher for the synergic treatment than for the control. We conclude that BO combined with EVOO could be an excellent food supplement for human consumption.

## 1. Introduction

*Mauritia flexuosa* L.f., regionally known as buriti, is a palm species (Arecaceae) distributed in tropical South America, especially in wetlands. The fruit has a thick, oily pulp, with an orange color attributed to its contents of lipids and carotenoids (β-carotene), which are the main antioxidants present. The fruit also contains fibers (22.18 g/100 g), potassium (218 mg/100 g), calcium (80.49 mg/100 g), and iron (1.88 mg/100 g) [1,2,3]. The fiber content can indicate it as an ally in intestinal health, disease prevention, and weight loss aid [2].

It is mentioned that buriti is considered a functional food precisely because of its vast nutritional composition, especially when we analyze its oil, due to the preventive role for several diseases related to oxidative stress [3]. The oil extracted from the buriti pulp is utilized by the cosmetics industry for products such as sun creams and creams for treating burns and preventing early skin aging due to its cicatrizing and antiaging action, which is attributed to the antioxidants (carotenoids and tocopherols) that act by eliminating free radicals [4,5].

Regarding its lipidic content, buriti oil presents a high oleic acid content (65.6%), followed by palmitic acid (19.2%), and smaller fractions of the fatty acids linolenic, linoleic, stearic, and myristic. This composition resembles olive oil, which indicates its high nutritional quality since the oleic fatty acid is protective against diseases such as cardiovascular and inflammatory diseases [6].

Furthermore, it has relevant antioxidant compounds, such as carotenoids and tocopherols, which prevent oxidative reactions in both food and the human body [7]. Of the carotenoids, β-carotene represents the main fraction, reaching 70%. This carotenoid is responsible for protecting the oil from oxidative damage because it interacts with free radicals and reactive oxygen species. In addition, they play a protective role in developing of some types of cancer, as they protect cells and tissues from oxidative damage [7,8]. Regarding tocopherols content, buriti oil has around 1169 µg/g, with α-tocopherol representing its main fraction. [2].

Vegetable oils tend to possess nutritional properties that promote the maintenance of health and, thus, can even help treat diseases because their compositions include fatty acids and antioxidants. It is reported that antioxidant components, such as carotenoids, tocopherols, and phenolic compounds, can act in synergy in foods and the organism through various mechanisms, many of which are yet to be unraveled [9,10,11,12].

Inflammation is a process directly related to oxidative stress and when uncontrolled, can lead to the development and progression of other disorders, e.g., cardiac and intestinal diseases [13,14]. Fatty acids and antioxidants have been shown to control the development of inflammation, especially polyunsaturated fatty acids, which reduce the levels of proinflammatory cytokines and also decrease clotting cascades related to platelet damage [3,6,15].

Therefore, our work had the objective of characterizing the crude oil of the buriti pulp and analyzing the effect of its supplementation for 90 days on the metabolism of Swiss mice in the search for a new option of edible vegetable oil that promotes the maintenance of health and the prevention of diseases.

## 2. Materials and Methods

### 2.1. Raw Materials

The crude oil extracted from the buriti pulp was acquired from Citróleo Group™, produced by the cold pressing of the ripe fruits of *Mauritia flexuosa*. The extra virgin olive oil was acquired from Andorinha Portugal^®^.

### 2.2. Fatty-Acid Profile and Nutritional Quality of Buriti Oil

The fatty acids were esterified according to the method adapted from Maya and Rodriguez-Amaya [16]. The methyl esters of the fatty acids were analyzed by gas chromatography (GC 2010, Shimadzu, Kyoto, Japan) to obtain their peaks. We utilized a flame ionization detector (FID) and a capillary column (BPX-70, internal diameter of 0.25 mm, 30 m long, and 0.25 mm thick film). The temperature of the injector and the detector was 250 °C. The initial temperature of the column was kept at 80 °C for 3 min and then increased by 10 °C/min until reaching 140 °C, followed by an increase to 240 °C/min for 5 min.

The individual peaks of the FAMEs (fatty-acid methyl esters) were identified by comparing their relative retention times with the standard of 37 FAMEs (Supelco C22, 99% pure). Based on the profile of fatty acids, we evaluated the following indices related to the nutritional quality of buriti oil:

1. Atherogenic index [17]:
  (1) $$\mathrm{Atherogenic}\;\mathrm{index}=\frac{C_{12:0}+4{\;x\;C}_{14:0}+C_{16:0}}{\;\Sigma \;\mathrm{MUFA}+\;\Sigma \;n6+\;\Sigma \;n3}$$
2. Thrombogenic index [17]:
  (2) $$\mathrm{Thrombogenic}\;\mathrm{index}=\frac{C_{14:0}+C_{16:0}+C_{18:0}}{0.5\times \;\Sigma \;\mathrm{MUFA}+0.5\times \;\Sigma \;n6+3\times \;\Sigma \;n3+\left(n3/n6\right)}$$
3. Hypocholesterolemic/hypercholesterolemic (HH) ratio [18]
  (3) $$\mathrm{HH}\;\mathrm{ratio}\;=\frac{C_{18:1\mathrm{cis}9}+C_{18:2n6}+C_{20:4n6}+C_{18:3n3}+C_{20:5n3}+C_{22:6n3}}{C_{14:0}+C_{16:0}}$$

### 2.3. Analyses of Quality and Identity of the Buriti Oil

We determined the indices of acidity (AOCS, 1990—method Ca 5a-40), peroxide (AOCS, 1990—method Cd 8-53), refraction (AOCS, 1990—method Cc 7-25), iodine (AOCS, 1990—method Cd 1-25), saponification (AOCS, 1990—method Cd 3-25), and relative density (AOCS, 1990—method Cc 10a-25) [19].

The acidity index was determined by adding a solution of ether alcohol (1:1), neutralizing the oil, and phenolphthalein as a color-changing indicator. As a titrant, we utilized 0.1N KOH until the pink color appeared, and the results were expressed in mgKOH/g.

The peroxide index was obtained by adding to the oil 5.0 mL of acetic acid–chloroform (3:2), 0.1 mL of a saturated solution of potassium iodide, and 0.1 mL of soluble starch (1%) as an indicator of color change, followed by incubation in the dark. Titration was performed with a solution of sodium thiosulfate (0.01N), and the results were expressed in MeqO_2_/kg.

The refraction index was obtained with a refractometer, Abbé (RL3, Tecnal, Ourinhos, Brazil), calibrated with distilled water, with a refraction index of 1.3330, at 27 °C, with the temperature corrected to 40 °C.

The iodine index was obtained by adding carbon tetrachloride and Wijs solution to the oil. As a titrant, we utilized standardized sodium thiosulfate until the color changed from black to pink, and the results were expressed in gI_2_/100 g.

The saponification index was determined by adding an alcoholic solution of KOH at 4% to the sample and performing reflux for 1 h. Next, phenolphthalein was added as a color-changing indicator, titration was performed with HCl (0.5 N) until the color changed, and the results were expressed in mgKOH/g.

The relative density was obtained using the pycnometer method, with the previous taring in an oven at 105 °C. Next, water at 20–23 °C was added, and the mixture was placed in a bath under a constant temperature (25 ± 0.1 °C). After 30 min, the water level was adjusted and weighed on an analytical balance. The same procedure was performed with the oil, and the results are expressed in milligrams per milliliter.

### 2.4. Optical Analyses of Buriti Oil: UV–Vis Absorption and Fluorescence Emission—Excitation Matrix

Buriti oil samples were diluted in hexane (spectroscopic grade, 99.9%) at 5 g/L. We utilized a quartz cuvette with a 10 mm optical path and four polished sides for optical measurements. The UV–vis absorption measurements were performed using a spectrophotometer (Lambda 265 UV/Vis, Perkin Elmer, Waltham, USA). The UV–vis absorption spectra were collected in the 200–600 nm range.

The fluorescence map (excitation/emission) of the oil was obtained utilizing a bench spectrofluorometer, FS-2 (Scinco, Seoul, Korea). The fluorescence excitation/emission matrices were obtained by exciting the samples in the 250–400 nm range, with steps of 5 nm, and the emission was collected between 250 and 600 nm, with a 1 nm resolution. For all tests, the excitation and emission slits were set at 5 nm, and all measurements were performed at room temperature.

### 2.5. Oxidative Stability: Rancimat Test

The oxidative stability was determined by obtaining the induction period (IP) through the Rancimat test, following the EM 14,112 method, by utilizing the Rancimat equipment (893 Professional Biodiesel Rancimat, Metrohm, São Paulo, Brazil). The analysis was performed by adding 3 g of oil without a solution for a reaction in a glass vase at 110 °C; then, the mixture was analyzed under a constant airflow of 10 L/h, passing through the samples and then flowing into a measuring recipient containing 50 mL of deionized water, where the conductivity generated by volatile products during oil degradation was measured as a function of time [20].

### 2.6. Thermic Analyses: Thermogravimetry/Derived Thermogravimetry (TG/DTG) and Differential Scanning Calorimetry (DSC)

The TG/DTG curves of buriti oil were obtained using a thermic analysis system (TGA Q50 of TA Instruments), with approximately 5.0 mg of sample in an inert nitrogen atmosphere, with a flow of 60 mL min^−1^ in an oven and 50 mL min^−1^ on a balance, and with heating at a rate of 10 °C min^−1^, to temperatures varying from ambient to 700 °C, using platinum crucibles for the samples.

The DSC curves were obtained in a DSC Q20 of TA Instruments coupled to a refrigerator system, RCS 90 (Refrigerated Cooling System), utilizing approximately 2.3 mg of sample, aluminium crucibles, and a similar empty crucible as a reference, in an inert nitrogen atmosphere with a flow of 50 mL min^−1^. After inserting the sample, the cell temperature was balanced at 60 °C, following an isotherm for 10 min. Then, the sample was cooled down to –60 °C at a cooling rate of 5 °C min^−1^, followed by a new isotherm for 10 min. Last, the sample was heated to 60 °C at 5 °C min ^−1^. The total analysis time was 70 min.

### 2.7. Antioxidant Activity: ORAC (Oxygen Radical Absorbance Capacity) Lipophilic, ABTS (2,2′-Azino-Bis (3-Ethylbenzothiazoline-6-Sulfonic Acid) and FRAP (Ferric Reducing Antioxidant Power) Methods

The ORAC lipophilic method was used based on Ou et al. [21], diluting the oil in a solution of randomly methylated 7-cyclodextrin (RMCD) (RMCD Trappsol^®^) at 7%, which was performed in aqueous acetone at 50% (v/v). After aliquoting the samples diluted in dark microplates, fluorescein and 2,2-azobis(2-amidinopropane) dihydrochloride (AAPH) were diluted in phosphate buffer (pH 7.4) and were rapidly added to the plate. The microplate was read in a reader with fluorescent filters, with an excitation wavelength of 485 nm and emission wavelength of 520 nm, and the fluorescence was read every min for 80 min. The area under the curve was calculated as described by Dávalos et al. [22]. The results were expressed in Trolox equivalent (TE) micromoles per 100 mL.

The antioxidant activity was determined by the ABTS method according to Rufino et al. [23]. An ABTS cationic solution was prepared by mixing 5 mL of ABTS (7 mM) and 88 µL of potassium persulfate solution (150 mM). The solution was reacted for 16 h at ambient temperature and in the absence of light. After the ABTS·+ radical was formed, we added ethanol until we obtained an absorbance value of 0.600 (±0.05) at 754 nm. The absorbance of the samples was determined at ambient temperature after 6 min of reaction. As a standard, we utilized Trolox as a reference antioxidant, and the results were expressed in micromoles of TE per 100 mL.

The FRAP method was used to determine the capacity for ferric reduction of the methanolic extract [24]. The FRAP reagent was prepared in the dark, using acetate buffer at 0.3 M (pH 3.6), TPTZ (10 mM) in a chloride acid solution (40 mM), and FeCl3 (20 mM) (10:1:1). The samples or standard solution (Trolox), water and FRAP reagent were mixed and incubated in an oven for 30 min at 37 °C. The absorbance of the samples and the standard Trolox curve at 595 nm was read, and the results were expressed in micromoles of TE per 100 mL.

### 2.8. Total Phenols (or Phenolic Compounds)

The total phenolics were quantified using the Folin–Ciocalteu method adapted from Swain and Hills [25] using microplates, and the absorbance was detected at 795 nm in a spectrophotometer (Thermo Scientific Multiskan GO, Thermo Fisher Scientific Corporation, Dubai, United Arab Emirates) after 90 min of rest. The results were expressed in milligrams of gallic acid equivalent (GAE) per 100 mL.

### 2.9. β-Carotene

To determine the β-carotene, we utilized the method of Maldonade et al. [26] to form an ether extract and performed absorbance readings in a spectrophotometer (450 nm). We utilized a standard of β-carotene (99% purity). The results were expressed in milligrams per 100 g of oil.

### 2.10. Coloration

The oil color was analyzed using the colorimeter (CM-2300d, Konica Minolta, Ramsey, NJ, USA), which expresses results on the CIE L*a*b* scale, where L* indicates the lightness, a* represents the band of red (+a) to green color (−a), and b* represents the band of yellow (+b) to blue color (−b). We obtained the hue and chroma angle indices from the results as established by Minolta Corporation [27].

### 2.11. Experimental Design

The experiment was developed according to the ethical principles of Law no. 11,794 of 8 October 2008, of the Decree no. 6899 of 15 July 2009, and the rules established by the National Council of Control of Animal Experimentation (CONCEA), and was approved by the Ethics Commission on the Use of Animals (CEUA) of UFMS (no. 1055/2019) (Annex 1).

We utilized 108 male, adult, 12-week-old Swiss mice (*Mus musculus*) obtained from the Biotherium of UFMS. The animals had a 7-day adaptation period in cages with a maximum of 4 individuals/cage. In the adaptation and experimental phase, the animals were kept in a quiet room under controlled temperature (23 ± 1 °C) and a 12 h of light/dark cycle, with access to food and water ad libitum.

#### 2.11.1. Animal Groups

**Figure 1:**
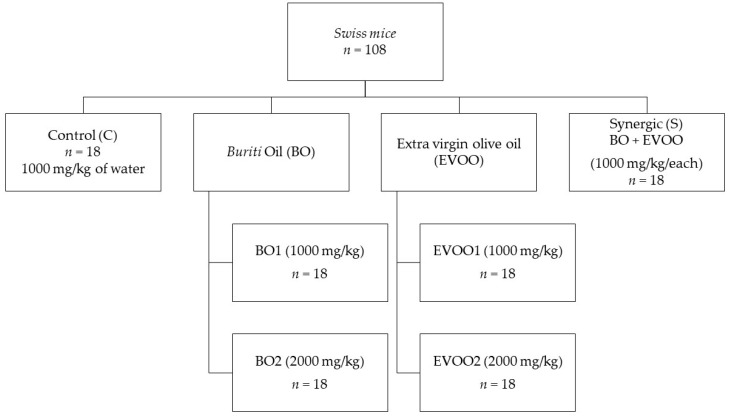
Intervention protocol for the supplementation with buriti oil. C, control group (without lipidic supplementation); EVOO1, extra virgin olive oil (1000 mg/kg); EVOO2, extra virgin olive oil (2000 mg/kg); BO1, buriti oil (1000 mg/kg); BO2, buriti oil (2000 mg/kg); Synergic, EVOO1 + BO1.

The animals were randomly split into six groups: a control group (receiving water at the dose of 1000 mg/kg), buriti oil group 1 (BO1) (receiving buriti oil at a dose of 1000 mg/kg), buriti oil group 2 (BO2) (receiving buriti oil at a dose of 2000 mg/kg), extra virgin olive oil 1 (EVOO1) (receiving extra virgin olive oil at a dose of 1000 mg/kg), extra virgin olive oil 2 (EVOO2) (receiving extra virgin olive oil at a dose of 2000 mg/kg), and synergic group (receiving a mix of BO1 and EVOO1), as shown in Figure 1 (adapted from Figueiredo et al. [28] and Silva et al. [29]).

The doses were administered daily via gavage during the 90 days of supplementation, being adjusted weekly to the weight of each animal. All the groups received standard commercial animal feed (Nuvital^®^). We weighed the animals weekly to verify the weekly food consumption.

#### 2.11.2. Euthanasia and Autopsy Sampling

Before euthanasia, the animals were made to fast for 8 h and provided only water. They were anesthetized with isoflurane for the procedure and euthanized by exsanguination. The visceral fats (epidydimal, mesenteric, retroperitoneal, and perirenal) and the liver were removed and weighed on a semianalytical electronic balance (Bel Diagnóstica^®^), and the values are expressed in grams, adapted from Chau et al. [30]. The adiposity index was calculated according to the formula of Taylor and Phillips [31].

#### 2.11.3. Serum Parameters

After collecting blood from the posterior vena cava, the blood was centrifuged at 17,000 rpm for 20 min in a centrifuge (Fanem^®^) to obtain the serum. An aliquot of 200 µL of serum was stored in an Eppendorf tube at −18 °C. We determined the levels of total cholesterol, high-density lipoprotein cholesterol (HDL-c), non-HDL-c, triglycerides, and glucose, utilizing the enzymatic–colorimetric method and spectrophotometric measurements [32,33,34].

#### 2.11.4. Histology of the Liver

The removed liver was stored in collector pots with formaldehyde at 10% for histological analyses. An experienced pathologist undertook the histological analyses in a blinded fashion. The analyses of the treatment effects on hepatocytes were performed utilizing the classification system of Kleiner et al. [35], assessing the Degree of Hepatic Steatosis (<5%, 5 to 33%, 33 to 66%, or >66%); Microvesicular Steatosis (absent or present); Lobular Inflammation (absent, <1 focus/field, 2 to 4 foci/field or >4 foci/field); Baloonization (absent, few cells or many cells); Mallory Hyaline (absent or present); Glucogened Nucleus (none/rare or a few); and Apoptosis (absent or present).

#### 2.11.5. Cytokines

The serum concentrations of interleukin-6 (IL-6) adipokines, monocyte chemoattractant protein-1 (MCP-1), tumor necrosis factor α (TNF-α), total plasminogen activator inhibitor-1 (PAI-1), insulin, leptin, and resistin were measured using the kit MILLIPLEX^®^ MAP MOUSE MADKMAG-71K (Merck Sigma-Aldrich^®^ Millipore, Billerica, MA, USA). Therefore, we separated the serum by centrifugation, subjected it to a vortex for 30 s and centrifuged it (6000 rpm for 10 min). Next, we spread 10 µL of the serum of each animal on a plate with 96 wells with 10 µL of assay buffer solution and 25 µL of a solution containing seven adipokines. We also prepared the white, standard, and control parameters (Milliplex MAP kit, Billerica, MA, USA). We read the plate in the Luminex^®^ using the software MAGPIX^®^, and the concentration values are expressed in picograms per milliliter and milligrams per milliliter.

#### 2.11.6. Statistical Analyses

The physical–chemical analyses were conducted in triplicate, and the results are expressed as the mean ± standard deviation of the mean (DP). The biological experiment results are expressed as the mean ± standard error of the mean (SEM). Analysis of variance (ANOVA) was used to compare groups, followed by Tukey’s post hoc or Dunn’s test when differences occurred. We utilized the chi-square test to evaluate the associations in the histological analyses, followed by Bonferroni corrections, using the statistical program Bioestat 5.0. The adopted significance level was *p* < 0.05.

## 3. Results and Discussion

### 3.1. Profile of Fatty Acids and Nutritional Quality of Buriti Oil

We identified twenty-one fatty acids in buriti oil, of which five showed values above 0.30%, as described in Table 1.

Among the fatty acids found, buriti oil showed the highest amounts of monounsaturated, among which the oleic fatty acid was the one that represented the main fraction (76.38%). Other acids found were the fatty acid palmitic (17.86%) and minor fractions of stearic fatty acid (1.07%), α-linolenic (0.83%), and γ-linolenic (0.37%).

Our results corroborate those found for buriti oil in previous studies, that reported in buriti oil the fatty acids oleic (65.60–73.05%), followed by palmitic (17.35–22.18%) [7,36,37]. Small variations found between studies may occur due to genetic factors of the species, environmental conditions, and even the analytical techniques used [7].

Furthermore, its composition is similar to that of olive oil, which presents oleic acid as the main fatty acid fraction (74.70%), followed by palmitic (12.20%), linoleic (8.50%), stearic (2.40%), and linolenic (0.60%) acid [38]. That indicates that buriti oil has an excellent lipidic profile regarding its high oleic acid content.

Vegetable oils with high oleic acid contents are less susceptible to oxidative reactions than oils with high contents of polyunsaturated fatty acids, especially linoleic fatty acid and, therefore, more stable [7,36]. Additionally, vegetable oils similar in profile to buriti oil can be alternatives for industrial use, such as margarine [37].

It is worth pointing out that oleic acid is also of high relevance for health because it is essential for the formation of hormones and preventing oxidative stress by stimulating the production of anti-inflammatory mediators, helping to protect against cardiovascular diseases, for example [39,40,41].

From the oil’s profile of fatty acids, we calculated the indices of nutritional quality (Table 2), indicators for predicting the possible risk for developing cardiovascular diseases [17].

There is a gap in the values for those indicators described in the legislation; thus, they are recommended to be the lowest possible [17]. Therefore, such indicators should be utilized in a manner complementary to other analyses because, in isolation, they do not represent the real biological action in animal models [42,43].

### 3.2. Analyses of Quality and Identity of the Buriti Oil

Vegetable oils should have acceptable nutritional and chemical quality, as defined by quality and identity indicators, which are also utilized to screen for possible adulterations [42]. In Table 3, we describe the results obtained for buriti oil.

Buriti oil presented an acidity index above the limit established by the Codex Alimentarius [44] for cold-pressed crude vegetable oils (<4 mgKOH/g) is superior to that reported in the literature for the same oil as in the study by Pardauil et al. [39] (2.67 mgKOH/g) and Freitas et al. [36] (3.99 mgKOH/g). That could be due to the extraction process (cold pressing) retaining organic compounds, such as free fatty acids [45].

The peroxide index is another indicator related to the quality of vegetable oils; it identifies the presence or absence of hydroperoxides formed in the initial oxidation steps [46]. In our study, buriti oil remained under the limit defined for this indicator (15 mEO2/kg) for crude and cold-pressed oils, indicating that it was not oxidized [44].

This result can be attributed to the antioxidant components present in buriti oil, such as phenolic compounds, carotenoids, and tocopherols, which help prevent oxidative events. The result found is lower than that described by the literature for the same oil, with values of 4.03 mEqO2/kg [39] and 6.86 mEqO2/kg [39].

The refraction index we determined for buriti oil was similar to the indices reported in the literature (1.46–1.47) [36,47]. This indicator is related to the viscosity, indicating deterioration because deteriorated oils become more viscous [46].

The iodide index is related to the unsaturation degree. It is a relevant indicator for determining the oil’s vulnerability to oxidation because the higher the degree of unsaturation, the more predisposed it is to oxidation [40,48]. For buriti oil, we detected an index similar to that previously described (81.10 to 90 gI_2_/100 g) [47].

For the saponification index, our result was within the limit established by the Codex Alimentarius [44] (<200 mgKOH/g). Values above this limit indicate the presence of higher quantities of fatty acids of low molecular weight [48].

For the relative density, our result was similar to that reported in the literature (0.90 to 0.92 mg/mL) [45,47].

### 3.3. Optical Analyses: UV–Vis Absorption and Emission—Excitation Fluorescence Matrix

**Figure 2:**
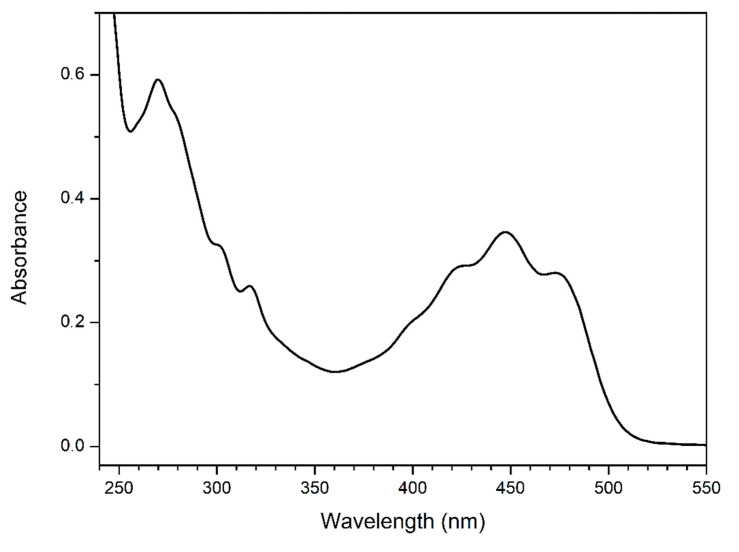
UV–vis absorption spectra of buriti oil diluted in hexane at 5 g/L.

Figure 2 shows the UV–vis absorption spectra of buriti oil and indicates that it presented absorption in the UV range of 250–350 nm that could be attributed to the $\pi -\pi^{*}$ and $n-\pi^{*}\;$electronic transitions originating from C=C and C=O, respectively, of fatty acids and their oxidative derivatives [49,50], as well as to the contribution of tocopherols, which present a strong absorption below 300 nm [51,52]. Regarding UV absorption, we also observed an absorption band in the range of 370-520 nm, which was attributed to the presence of carotenoids [53,54].

Therefore, our results for the UV–vis spectrum indicate the presence of the band of carotenoids, with absorption in the blue-greenish range (375–520 nm), implying that they are the main natural pigments present in buriti oil.

**Figure 3:**
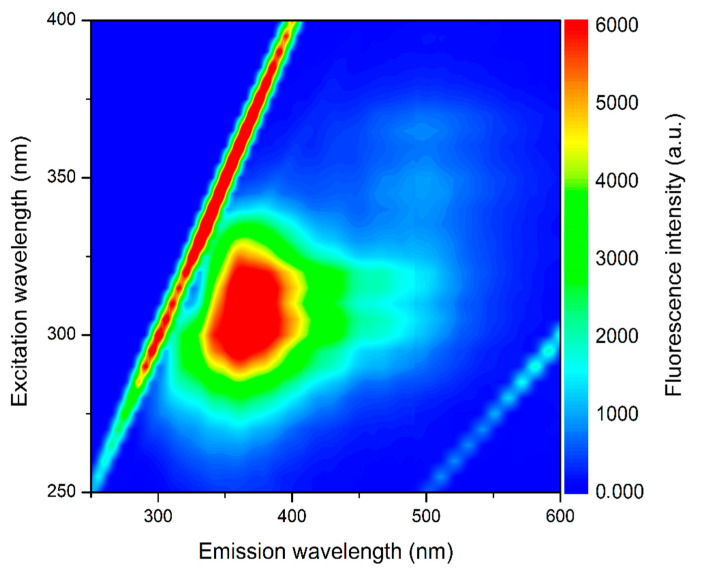
Emission–excitation map of buriti oil diluted in hexane at 5 g/L.

Figure 3 shows the emission–excitation fluorescence matrix for buriti oil, whereby it is possible to observe high-intensity emission between 300–500 nm when excited in the 250–350 nm range, presenting a maximum of fluorescence with the excitation/emission at around 310/350 nm. The observed emission band can be ascribed to the endogenous fluorescent compounds in vegetable oils, such as tocopherols and carotenoids [50]. It is well known that tocopherols present a relatively intense emission band in the 300–350 nm range when excited between 270 and 310 nm, as observed by Sikorska et al. for edible oils [55].

### 3.4. Oxidative Stability: Rancimat

The Rancimat test can provide the IP of oils, which directly indicates their oxidation and quality during a heating phase, whereby the lower the quantity of polyunsaturated fatty acids, the higher the IP value [49].

**Figure 4:**
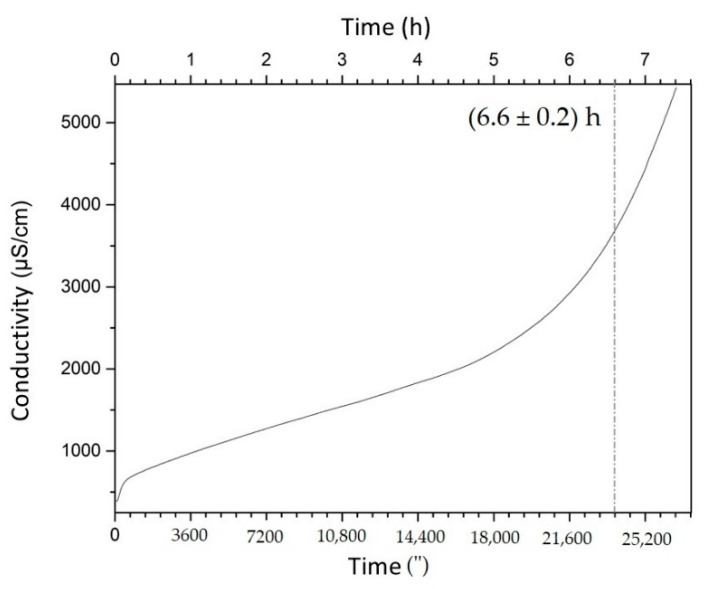
Electric conductivity versus time determined by the Rancimat method for buriti oil.

In our study, buriti oil showed an IP of 6.60 h (Figure 4), lower than the observed value for olive oil (9.02 h) but superior to the values for other vegetable oils, such as sunflower (2.41 h) and rapeseed (4.95 h) [49].

### 3.5. Thermic Analyses: TG/DTG and DSC

**Figure 5:**
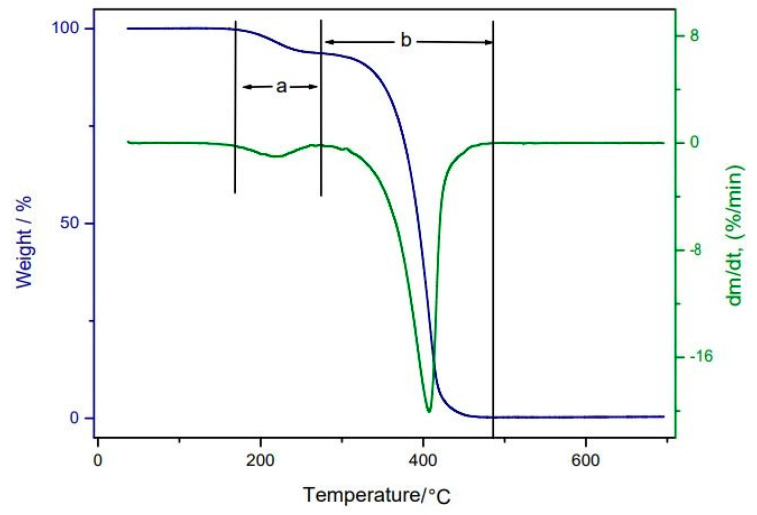
TG/DTG curves for buriti oil; a = first step; b = second step.

The TG/DTG curves for buriti oil indicate that the thermal decomposition occurred in two well-defined steps, including the first mass loss approximately between 174 and 276 °C, attributed to moisture loss and the volatilization of relatively low molecular weight compounds (aldehydes and short-chain fatty acids). The second mass loss occurred between 276 and 479 °C, which can be attributed to the decomposition of the long-chain fatty acids, which lose mass at different temperatures [39,56]. In this event, we observed a mass loss of 93.38% and the formation of a residue of ashes of 0.3038%, as shown in Figure 5.

**Figure 6:**
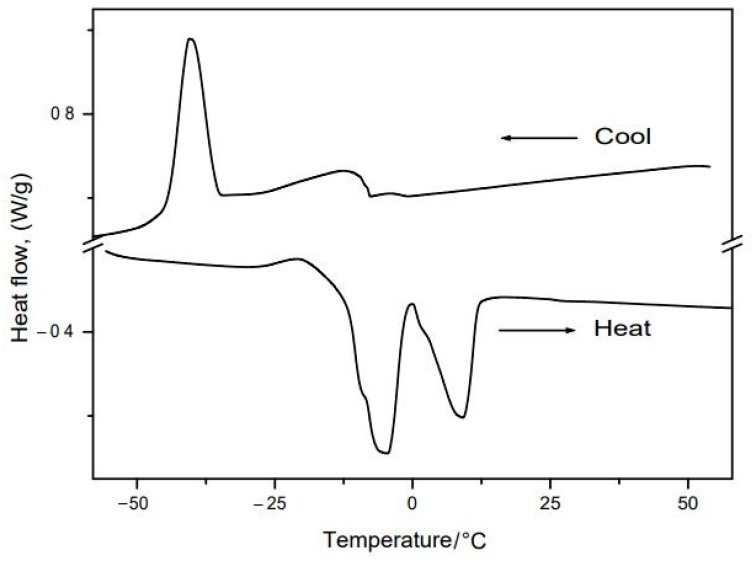
DSC curves of buriti oil with fusion point (**bottom**) and crystallization point (**top**).

The DSC curves for buriti oil are presented in Figure 6. We observed two endothermic events in the first cycle (cooling) relative to the crystallization temperatures of mixtures of saturated and unsaturated fatty acids, respectively. The temperatures were calculated with the help of the onset point tool and the absorbed and liberated energies (ΔH), with the integration tool for the peak area, both in the software Universal Analysis (TA Instruments).

The first peak begins at around −7.8 °C, with a maximum peak close to −11.4 °C, and ΔH of 7.992 J·g^−1^. The second, sharper, well-defined peak begins at around −35.7 °C, with a maximum peak at −40.37 °C and ΔH of 26.59 J·g^−1^. According to Alexandre et al. [57], such peak characteristics are related to unsaturated fatty acids in higher quantities, as can be verified in Table 1.

Regarding the heating curve (second cycle), we observed the fusion points of the unsaturated and saturated fatty acids, respectively. The first peak occurs at around −17.6 °C, with a maximum peak at −5.3 °C and ΔH of 38.43 J·g^−1^, and the second, at around 0.1 °C, with a maximum at 8.85 °C, and ΔH of 22.38 J·g^−1^. That displacement of the initial temperatures between the events in different cycles (cooling x heating) is to be expected due to the different kinetics of the physical processes.

The literature shows that vegetable oils with higher contents of unsaturated fatty acids present crystallization points closer to −50 °C due to more energy being needed to organize the structure to crystallize [58].

The results obtained by Freitas et al. [35] for the DSC curves show similar temperatures for the fusion and crystallization of buriti oil because of its content of triacylglycerols, and both points occur within temperature ranges rather than at specific temperatures.

### 3.6. Antioxidant Activity: ORAC, ABTS, and FRAP Methods

The antioxidant activity of buriti oil assessed through ORAC, ABTS, and FRAP methods is described below (Table 4).

Each method determines the antioxidant activity of a sample in one mode. The ORAC method, for example, utilizes the transference of hydrogen to determine how much a compound can protect against the degradation of fluorescein; the higher the antioxidant capacity, the lower the degradation [7,59].

In turn, the ABTS method measures the antioxidant capacity of a compound based on the transference of hydrogen and electrons, and the FRAP method measures the reduction capacity of an antioxidant through its reaction with the iron tripyridyl triazine complex, resulting in a colored iron tripyridyl triazine complex [4,59].

For the three methods, our results were lower than those already reported for buriti oil, which could have various causes, such as the solvent type, processing, and even environmental factors [7,60]. Despite that, it was possible to observe that buriti oil showed antioxidant potential attributed to its antioxidant components, such as carotenoids and total phenolic compounds.

### 3.7. Total Phenolic Compounds

Phenolic compounds are antioxidants found in relevant foods because they prevent oxidative stress and reduce inflammatory processes and bacterial infections. Furthermore, they can protect other components present in foods, such as unsaturated fatty acids and tocopherols [7,61].

In our study, the content of total phenolic compounds found in buriti oil (21.57 ± 0.83 mgAGE/100 g) was superior to that reported in the literature (10.70 mgAGE/100 g) [6,7]. Variations in content can occur due to differences in the environmental conditions of each collected fruit [7].

### 3.8. β-Carotene

We detected 51.01 ± 1.46 mg/100 g of β-carotene in buriti oil. However, our result was inferior to the 71 mg/100 g [6] and 78.16 mg/100 g [7] reported by other authors for the same oil. The buriti oil had β-carotene as its main carotenoid fraction, reaching 70% of your composition [7].

This result, associated with the other antioxidants present, reflected its stability, as highlighted above, and may reflect on health effects since such carotenoid acts by protecting cells from the action of free radicals [7,8].

### 3.9. Coloration

Table 5 shows the colorimetric parameters of buriti oil, where we observed that the L* (lightness) was darker, as also observed for its saturation (C*). For a* (red axe (+)/green (−)), the result was positive, indicating a more reddish color, and for b* (yellow axe (+)/blue (−)), the result was also positive, indicating a more yellowish color.

Buriti oil presented a yellowish color based on the observation of the hue angle (°), indicative of tone. such results can be attributed to the presence of the carotenoids common in the composition of buriti oil and representing the primary pigments, especially β-carotene, as previously reported. 

### 3.10. Experimental Design

#### 3.10.1. Weight Gain and Food Ingestion

The animals were weighed weekly to monitor weight gain. Through the results, we did not observe significant differences between groups for final weight and food intake (Table 6), a result similar to that reported by Aquino et al. [62], in which Wistar rats supplemented with buriti oil did not show significant differences and did not differ from the other groups receiving other oils as supplementation (soybean oil and refined buriti oil).

Therefore, we observed that the different types of oils and in different doses were not responsible for alterations in the weight and consumption of food for the animals after 90 days of supplementation.

#### 3.10.2. Weights of Liver and Visceral Fats, and Adiposity Index

Regarding the liver weight and adiposity index, we did not detect significant differences between groups (Table 7).

Lipid supplements at different concentrations caused only minor changes in liver weight and fat sites. Only the mesenteric and perirenal fats presented significant differences between groups for the visceral fats. For mesenteric fat, the EVOO1 group differed from the EVOO2 group, while for the perirenal, the EVOO2 group differed from the synergic group. Regarding the adiposity index, there was no statistically significant difference between groups, and the low adiposity index found can be attributed to the high content of unsaturated fatty acids that are responsible for helping to reduce body fat [41].

#### 3.10.3. Biochemical Parameters

Regarding the biochemical parameters, the contents of triglycerides, fastening glucose, and total cholesterol did not present significant differences between groups, as described in Table 8. The fractions HDL-c and non-HDL-c presented significant differences.

For HDL-c, we observed that the control group, with the highest value, differed from the synergic (*p* = 0.03) and BO2 (*p* = 0.037) groups, while the EVOO2 group, also with a low value, differed from the synergic (*p* = 0.04) and BO2 (*p* = 0.05) groups. In the non-HDL-c fraction, we observed that the control group, with the highest value, differed from the synergic (*p* = 0.001) and BO1 (*p* = 0.001) groups, while the EVOO1 group, also with a higher value, differed from the BO1 (*p* = 0.036) and synergic groups (*p* = 0.001).

It is possible to note that the synergic group presented the best relation between the cholesterol fractions because the higher the HDL-c content and the lower the non-HDL-c content, the more protective the supplementation was against cardiovascular diseases when we associate both results.

This result may have been due to combined fatty acids from buriti oil and olive oil. As the main fractions, both oils have monounsaturated fatty acids (such as oleic acid), which have been shown to prevent inflammatory diseases and show anti-atherogenic activity, besides controlling the glycemic profile [10,63,64].

The antioxidants present in both oils (BO + EVOO) may also have influenced the result because they can reduce the LDL-c content, prevent platelet aggregation, and increase the action of HDL-c, as described in the literature for these isolated components, also indicating its synergistic action [30,65,66].

#### 3.10.4. Hepatic Histology

Among the analyzed groups, only the BO1 group presented an animal with steatosis >5%, with no association between the presence of steatosis and the experimental groups being observed (*p* = 0.41). In another study, the consumption of buriti oil at the highest dose (100 mg) also induced steatosis in the animals [67]. However, it was important that the animals in this study were obese, which may have contributed to the result (which was not observed in our animal study).

However, in our study, the localization of the steatosis could not be analyzed due to the low frequency of the observations. Microvesicular steatosis was not observed in any of the analyzed groups; therefore, it was not possible to carry out an inferential statistical analysis (Table 9).

The absence of steatosis could be related to the antioxidants present in both oils, which protect against hepatic damage caused by different factors, such as proinflammatory cytokines including TNF-α and IL-6 [68,69].

Regarding lobular inflammation, most animals did not present foci independent from the experimental group (*p* = 0.09). By contrast, the association between the groups was significant in the ballooning data. The EVOO1 group presented a higher prevalence of ballooned cells than the control and EVOO2 groups; however, there was no difference between the other groups (*p* = 0.01).

Only the BO1 group presented Mallory hyaline in one animal, which was absent in all the other groups (*p* = 0.41). Additionally, we did not find an association between the groups and the presence of apoptosis (*p* = 0.40) or nuclear glycogenation (*p* = 0.41), as the latter was identified in only one animal of the AOE1 group.

#### 3.10.5. Quantification of Cytokines

**Figure 7:**
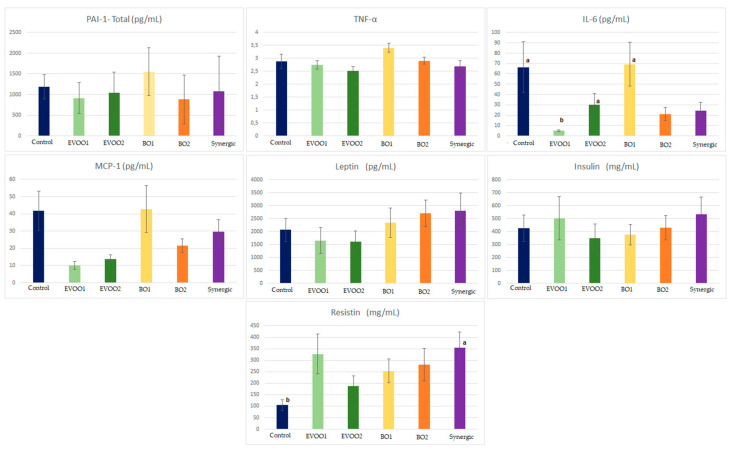
Concentration of cytokines in the serum after 90 days of supplementation. C, control group (without lipidic supplementation); EVOO1, extra virgin olive oil (1000 mg/kg); EVOO2, extra virgin olive oil (2000 mg/kg); BO1, crude buriti oil (1000 mg/kg); BO2, crude buriti oil (2000 mg/kg); S, synergic—BO1 + EVOO1. The values represent the mean ± standard error of the mean. For the total PAI-1 (*p* = 0.119), TNF-α (*p* = 0.063), MCP-1 (0.066), leptin (0.467), and insulin (0.583), there was no significant difference between the groups. ANOVA, followed by Tukey’s post hoc test. For IL-6 and resistin, the values differed between them (*p* = 0.003 and 0.025, respectively), according to ANOVA followed by Dunn’s post hoc test. Notes: ^a^: indicates the group with the lowest value for the analyzed parameter and that differed from the group with letter ^b^; ^b^: indicates the group with the highest value for the analyzed parameter and that differed from the group with letter ^a^.

The results for the total PAI-1, TNF-α, IL-6, MCP-1, leptin, insulin, and resistin can be observed below (Figure 7).

The adipose tissue is an endocrine organ capable of secreting adipokines and hormones that directly influence the development of inflammation. With an imbalance in the production of these substances, one can observe the development of an inflammatory condition and, thus, alterations, such as insulin resistance, steatosis, and cardiovascular diseases [70,71].

Thus, it is extremely important to verify the impact that diet and lifestyle habits influence the markers formed in adipose tissue, especially targeting the loss of weight that demonstrates to improve the concentrations of these parameters [72].

Among the markers analyzed in our study, only IL-6 and resistin demonstrated significant differences between the groups. In this case, IL-6 showed a significant difference (*p* = 0.003) in that the control, EVOO2, and BO1 groups differed from the EVOO1 group.

IL-6 is a proinflammatory cytokine related to the development of insulin resistance, especially observed in high concentrations in obese individuals [11,70,71,73]. Additionally, the excessive production of IL-6 can affect the liver’s metabolism due to the induction of the secretion of VLDL-c (very-low-density lipoprotein) [69].

It is possible to conclude from our results that olive oil at the lowest dose reduced the concentrations of IL-6 and, thus, could have helped to prevent its elevation in the synergic group. The literature reports that the richness of olive oil in antioxidants such as oleuropein and hydroxytyrosol can reduce pro-inflammatory cytokines and, thus, protect the organism from inflammation [74].

We observed a significant difference (*p* = 0.025) regarding resistin between the control and synergic groups. Resistin is an adipocytokine commonly found in the organism when inflammation is already present, and as IL-6 levels rise, resistin concentrations also increase [75].

However, in our study, we observed an opposite relationship: the EVOO1 group, with the lowest IL-6 level, showed one of the highest concentrations of resistin, while the control group presented high IL-6 levels and the lowest resistin concentrations.

In general, all the parameters we analyzed are correlated with the development or inhibition of alterations in the organism when adipose tissue increases. Despite relevant markers such as IL-6 and resistin showing significant differences, we did not detect significance associated with the preventive role of antioxidants and fatty acids in buriti oil and extra virgin olive oil for many parameters.

It is also observed that the EVOO1 group was the best possible against markers to igniters and that its association with buriti oil (synergistic group) had better results when compared to the group, getting the lowest dose of this oil (BO1), and thus indicating the joint action of the antioxidants present in the two oils.

## 4. Conclusions

In this in vivo study, we observed that the crude oil extracted from buriti pulp shows excellent oxidative stability for its bioactive compounds; supplementation with the oil did not interfere with food consumption and weight and did not cause significant histological alterations in the livers of animals fed with a normocaloric diet.

Furthermore, synergic supplementation in the animals (BO1 + EVOO1) showed the strongest relationship between HDL-c and non-HDL-c, indicating a possible cardioprotective effect. The association of buriti oil with olive oil also improved inflammatory parameters, such as IL-6, compared with the BO1 group.

Thus, we conclude that buriti oil is an excellent option for edible vegetable oil. Its consumption should be incentivized in addition to that of extra virgin olive oil, another source of antioxidants and monounsaturated fatty acids.

We highlight the importance of further studies on the impacts of consumption in animals consuming hypercaloric diets regarding overweight and its associated comorbidities.

## Figures and Tables

**Table 1:** Fatty acid profile (%) of buriti oil.

Fatty Acids	Buriti Oil (%)
**Saturated**	
Butyric, C4:0	0.09 ± 0.01
Myristic, C14:0	0.05 ± 0.01
Pentadecanoic, C15:0	0.02 ± 0.01
Palmitic, C16:0	17.86 ± 0.00
Heptadecanoic, C17:0	0.07 ± 0.02
Stearic, C18:0	1.07 ± 0.01
Arachidic, C20:0	0.08 ± 0.01
*∑ TOTAL*	*19.24*
**Monounsaturated**	
Palmitoleic, C16:1	0.18 ± 0.02
Cis-10-heptadecanoic, C17:1	0.05 ± 0.01
Oleic, C18:1	76.38 ± 0.03
Gadoleic, C20:1	0.03 ± 0.00
Erucic, C22:1	0.03 ± 0.01
Nervonic, C24:1	0.02 ± 0.01
*∑ TOTAL*	*76.69*
**Polyunsaturated**	
Linoleic, C18:2	0.01 ± 0.00
α-linolenic, C18:3	0.83 ± 0.01
γ-linolenic, C18:3	0.37 ± 0.02
Cis-11,14-eicosadienoic, C20:2	0.04 ± 0.01
Arachidonic, C20:4	0.02 ± 0.01
Eicosapentanoic, C20:5	0.03 ± 0.01
Cis-13,16-docosadienoic, C22:2	0.03 ± 0.01
Cis-4,7,10,13,16,19-docosahexaenoic, C22:6	0.02 ± 0.00
*∑ TOTAL*	*1.35*

The means were determined from triplicates. Values are expressed as the mean ± standard deviation of the mean.

**Table 2:** Indices of nutritional quality of buriti oil.

Indices	Buriti Oil
Atherogenic	0.23
Thrombogenic	0.15
Hypocholesterolemic/hypercholesterolemic	4.31

**Table 3:** Indices of quality and identity for the buriti oil.

Indices	Buriti Oil
Acidity (mgKOH/g)	4.70 ± 0.18
Peroxide (mEq_2_/kg)	2.13 ± 0.30
Refraction at 40 °C	1.47 ± 0.00
Iodide (gI_2_/100 g)	87.56 ± 0.77
Saponification (mgKOH/g)	188.62 ± 3.31
Relative density (mg/mL)	0.91 ± 0.00

The means were determined from triplicates. Values are expressed as the mean ± standard deviation of the mean.

**Table 4:** Antioxidant activity of buriti oil.

Parameters	Buriti Oil
ORAC (µmol TE/100 mL)	1.55 ± 0.03
ABTS (µmol TE/100 mL)	1758.02 ± 6.97
FRAP (µmol TE/100 mL)	164.86 ± 2.41

The means were determined from triplicates. Values are expressed as the mean ± standard deviation of the mean.

**Table 5:** Colorimetric parameters of buriti oil.

Parameters	Buriti Oil
L*	43.87 ± 0.00
C*	34.72 ± 0.00
*Hue (°)*	72.99 ± 0.00
a*	26.73 ± 0.00
b*	22.16 ± 0.00

The means were determined from triplicates. Values are expressed as the mean ± standard deviation of the mean. L*, lightness; C*, saturation; a*, red axe (=)/green (−) and b*, yellow axe (=)/blue (−).

**Table 6:** Final weight (g) of the animals after 90 days of supplementation and mean daily food ingestion (g/day).

Parameters	C (*n* = 12)	EVOO1 (*n* = 12)	EVOO2 (*n* = 12)	BO1 (*n* = 12)	BO2 (*n* = 12)	Synergic (*n* = 12)
Final weight	45.05 ± 1.38	45.16 ± 1.39	44.44 ± 1.33	47.77 ± 1.52	45.00 ± 0.88	46.61 ± 0.98
Daily food ingestion	5.85 ± 0.13	5.58 ± 0.03	5.72 ± 0.05	5.76 ± 0.06	5.83 ± 0.05	5.78 ± 0.08

Values are expressed as the mean ± standard error of the mean. C, control group (without lipidic supplementation); EVOO1, extra virgin olive oil (1000 mg/kg); EVOO2, extra virgin olive oil (2000 mg/kg); BO1, buriti oil (1000 mg/kg); BO, buriti oil (2000 mg/kg); Synergic, EVOO1 + BO1.

**Table 7:** Weights (g) of liver and visceral fats (g), and adiposity index (%).

Parameters	C (*n* = 12)	EVOO1 (*n* = 12)	EVOO2 (*n* = 12)	BO1 (*n* = 12)	BO2 (*n* = 12)	Synergic (*n* = 12)
Liver	1.82 ± 0.06	1.77 ± 0.06	1.84 ± 0.07	2.03 ± 0.11	1.74 ± 0.03	1.91 ± 0.06
Epidydimal	1.34 ± 0.13	1.52 ± 0.11	1.62 ± 0.14	1.62 ± 0.14	1.53 ± 0.13	1.76 ± 0.11
Mesenteric	0.75 ± 0.08 ^ab^	0.78 ± 0.09 ^b^	0.47 ± 0.04 ^a^	0.61 ± 0.06 ^ab^	0.67 ± 0.06 ^ab^	0.71 ± 0.05 ^ab^
Retroperitoneal	0.49 ± 0.04	0.56 ± 0.04	0.44 ± 0.04	0.60 ± 0.05	0.57 ± 0.04	0.60 ± 0.04
Perirenal	0.21 ± 0.02 ^ab^	0.22 ± 0.02 ^ab^	0.14 ± 0.01 ^a^	0.20 ± 0.01 ^ab^	0.20 ± 0.01 ^ab^	0.25 ± 0.02 ^b^
Adiposity index	6.32 ± 0.43	6.53 ± 0.38	5.50 ± 0.45	6.55 ± 0.35	6.70 ± 0.40	7.21 ± 0.33

C, control group (without lipidic supplementation); EVOO1, extra virgin olive oil (1000 mg/kg); EVOO2, extra virgin olive oil (2000 mg/kg); BO1, buriti oil (1000 mg/kg); BO2, buriti oil (2000 mg/kg); Synergic, EVOO1 + BO1. Values are expressed as the mean ± standard error of the mean. Different letters in the same line indicate significant differences in the comparison between groups (*p* < 0.05): one-way ANOVA with Tukey’s post hoc test. Notes: ^a^: indicates the group with the lowest value for the analyzed parameter and that differed from the group with letter ^b^; ^ab^: indicates the group that did not differ statistically from the groups with the highest and lowest value for the analyzed parameter (^a^ and ^b^); ^b^: indicates the group with the highest value for the analyzed parameter and that differed from the group with letter ^a^.

**Table 8:** Biochemical parameters (mg.dL^−1^) of the animals after 90 days of supplementation.

Parameters	C (*n* = 12)	EVOO1 (*n* = 12)	EVOO2 (*n* = 12)	BO1 (*n* = 12)	BO2 (*n* = 12)	Synergic (*n* = 12)
Triglycerides	210.57±12.30	171.62 ± 7.34	182.63 ± 8.50	194.61 ± 14.24	180.40 ± 7.58	182.18 ± 7.32
Glucose	172.38 ± 28.94	173.15 ± 15.87	163.85 ± 13.02	162.78 ± 14.97	180.85 ± 17.65	168.87 ± 15.90
Total cholesterol	1.59.47 ± 5.81	165.80 ± 8.40	150.18 ± 7.55	150.67 ± 7.51	160.46 ± 7.86	158.13 ± 4.75
HDL-c	91.90 ± 3.17 ^a^	92.05 ± 4.16 ^ab^	82.61 ± 4.97 ^a^	90.75 ± 5.05 ^ab^	101.48 ± 5.45 ^b^	106.85 ± 4.33 ^b^
Non-HDL-c	77.57 ± 3.59 ^b^	78.88 ± 6.40 ^b^	64.80 ± 5.22 ^ab^	59.90 ± 4.09 ^a^	62.33 ± 4.20 ^ab^	51.11 ± 2.04 ^a^

C, control group (without lipidic supplementation); EVOO1, extra virgin olive oil (1000 mg/kg); EVOO2, extra virgin olive oil (2000 mg/kg); BO1, buriti oil (1000 mg/kg); BO2, buriti oil (2000 mg/kg); Synergic, EVOO1 + BO1; HDL-c, high-density lipoprotein cholesterol. Values are expressed as the mean ± standard error of the mean. Different letters in the same line indicate significant differences in the comparison between groups (*p* < 0.05); one-way ANOVA followed by Tukey’s post hoc test. Notes: ^a^: indicates the group with the lowest value for the analyzed parameter and that differed from the group with letter ^b^; ^ab^: indicates the group that did not differ statistically from the groups with the highest and lowest value for the analyzed parameter (^a^ and ^b^); ^b^: indicates the group with the highest value for the analyzed parameter and that differed from the group with letter ^a^.

**Table 9:** Histopathological analyses of the liver with scores for hepatic steatosis, localization of steatosis, macrovesicular steatosis, lobular inflammation, ballooning, Mallory hyaline, apoptosis, and nuclear glycogenation of the animals after 90 days of supplementation.

Variables	C (*n* = 12)	EVOO1 (*n* = 12)	EVOO2 (*n* = 12)	BO1 (*n* = 12)	BO2 (*n* = 12)	Synergic (*n* = 12)
**Steatosis (*p* = 0.41)**						
<5%	100.00 (12)	100.00 (12)	100.00 (12)	91.70 (11)	100.00 (12)	100.00 (12)
5 a 33%	0.00 (0)	0.00 (0)	0.00 (0)	8.30 (1)	0.00 (0)	0.00 (0)
Localization of steatosis *						
Zone 1	0.00 (0)	0.00 (0)	0.00 (0)	8.30 (1)	0.00 (0)	8.30 (1)
Zone 3	0.00 (0)	8.30 (1)	0.00 (0)	8.30 (1)	0.00 (0)	0.00 (0)
Not applicable	100.00 (12)	91.70 (11)	100.00 (12)	83.30 (10)	100.00 (12)	91.70 (11)
Microvesicular steatosis *						
Absent	100.00 (12)	100.00 (12)	100.00 (12)	100.00 (12)	100.00 (12)	100.00 (12)
**Lobular inflammation** **(*p* = 0.09)**						
No focus	100.00 (12)	75.00 (9)	75.00 (9)	50.00 (6)	50.00 (6)	83.30 (10)
<2 foci/field	0.00 (0)	8.30 (1)	25.00 (3)	41.70(5)	41.70 (5)	16.70 (2)
2–4 foci/field	0.00 (0)	16.70 (2)	0.00 (0)	8.30 (1)	8.30 (1)	0.00 (0)
**Ballooning (*p* = 0.01)**						
Absent	58.30 (7) ^b^	8.30(1) ^a^	58.30(7) ^b^	33.30 (4) ^ab^	33.30 (4) ^ab^	25.00 (3) ^ab^
Few cells	41.70 (5)	58.30 (7)	41.70 (5)	25.00 (3)	66.70 (8)	58.30 (7)
Many cells	0.00 (0)	33.30 (4)	0.00 (0)	41.70 (5)	0.00 (0)	16.70 (2)
**Mallory hyaline (*p* = 0.41)**						
Absent	100.00 (12)	100.00 (12)	100.00 (12)	91.70 (11)	100.00 (12)	100.00 (12)
Rare	0.00 (0)	0.00 (0)	0.00 (0)	8.30 (1)	0.00 (0)	0.00 (0)
**Apoptosis (*p* = 0.40)**						
Absent	100.00 (12)	75.00 (9)	91.70 (11)	75.00 (9)	83.30 (10)	91.70 (11)
Rare hepatocytes	0.00 (0)	25.00 (3)	8.30 (1)	25.00 (3)	16.70 (2)	8.30 (1)
**Nuclear glycogenation** **(*p* = 0.41)**						
None to rare	100.00 (12)	91.70 (11)	100.00 (12)	100.00 (12)	100.00 (12)	100.00 (12)
Much	0.00 (0)	8.30 (1)	0.00 (0)	0.00 (0)	0.00 (0)	0.00 (0)

C, control group (without lipidic supplementation); EVOO1, extra virgin olive oil (1000 mg/kg); EVOO2, extra virgin olive oil (2000 mg/kg); BO1, buriti oil (1000 mg/kg); BO2, buriti oil (2000 mg/kg); Synergic, EVOO1 + BO1. Data are presented as the relative frequency (absolute frequency). p values were determined with chi-squared tests with Bonferroni corrections. Different letters in the same line indicate significant differences between groups. Notes: ^a^: indicates the group with the lowest value for the analyzed parameter and that differed from the group with letter ^b^; ^ab^: indicates the group that did not differ statistically from the groups with the highest and lowest value for the analyzed parameter (^a^ and ^b^); ^b^: indicates the group with the highest value for the analyzed parameter and that differed from the group with letter ^a^. * It was not possible to perform inferential analysis due to the distribution of the sampling size.
